# De novo familial adenomatous polyposis associated thyroid cancer with a c.2929delG frameshift deletion mutation in APC: a case report and literature review

**DOI:** 10.1186/s12957-023-02951-9

**Published:** 2023-03-02

**Authors:** Miaorong Xu, Yuyan Zheng, Zhongchao Zuo, Qin Zhou, Qun Deng, Jianwei Wang, Da Wang

**Affiliations:** Department of Colorectal Surgery and Oncology, Key Laboratory of Cancer Prevention and Intervention, Ministry of Education, The Second Affiliated Hospital, Zhejiang University School of Medicine, Jiefang Road 88Th, Hangzhou, 310009 Zhejiang Province People’s Republic of China

**Keywords:** APC, De novo, Familial adenomatous polyposis, Germline mutation, Thyroid cancer

## Abstract

**Background:**

Germline mutations in the APC gene located on chromosome 5q 21–22 can lead to familial adenomatous polyposis (FAP) and the development of colorectal cancer (CRC) if left untreated. As a rare extracolonic manifestation, thyroid cancer is diagnosed in about 2.6% of FAP patients. The genotype–phenotype correlation in FAP patients with thyroid cancer remains unclear.

**Case presentation:**

We present a 20-year-old female of FAP with thyroid cancer as the initial manifestation. The patient was asymptomatic and developed colon cancer liver metastases 2 years after the diagnosis of thyroid cancer. The patient underwent multiple surgical treatments in several organs, and regular colonoscopy with endoscopic polypectomy was performed. Genetic testing demonstrated the c.2929delG (p.Gly977Valfs*3) variant in exon 15 of the APC gene. This represents a previously undescribed APC mutation. This mutation causes loss of multiple structures on the APC gene including the 20-amino acid repeats, the EB1 binding domain, and the HDLG binding site, which may be pathogenic through β-catenin accumulation, cell cycle microtubule dysregulation, and tumor suppressor inactivation.

**Conclusions:**

We report a de novo FAP case with thyroid cancer presenting atypically aggressive features harboring a novel APC mutation and review APC germline mutations in patients with FAP-associated thyroid cancer.

## Background

Familial adenomatous polyposis (FAP) is an autosomal dominant syndrome characterized by the development of hundreds to thousands of colorectal adenomatous polyps, resulting in colorectal cancer (CRC) in the majority of patients by the age of 40–50 if not treated [1]. The estimated incidence of FAP is within 1:7,000 to 1:30,000 of live births [2]. Besides, FAP is one of the best-known genetic diseases and accounts for approximately 1% of all CRC cases [3]. In recent years, there is a growing recognition that FAP is not merely a disease restricted to the colon and rectum but a multisystem syndrome with both nonmalignant and malignant conditions of various organs [4]. Screening of patients and their family members with timely treatment of affected individuals has led to a 55% reduction in the occurrence of CRC at diagnosis of FAP, and an improvement in cumulative survival for all FAP patients [5–7]. Although FAP is a familial disease, 11–25% of FAP patients are reported to have de novo mutations in the adenomatous polyposis coli (APC) gene [8]. In addition, extracolonic manifestations may precede FAP diagnosis, for example, a study by Chenbhanich et al. showed that thyroid carcinoma (TC) diagnosis preceded the diagnosis of FAP in about one third of the patients [4]. It is therefore crucial to conduct an in-depth investigation of the clinical biological behavior of FAP.

Up to 90% of classic FAPs are caused by inactivating germline mutations in the APC gene [9], which encodes a tumor suppressor that participates in WNT signaling pathway [10]. Mutations in certain regions of the APC gene may correlate with the severity of the disease and the extracolonic phenotypes. For example, mutations in the 5′ end, in exon 9, and in the 3′ end of the gene cause a relatively less severe form of FAP [11]. In addition, congenital hypertrophy of the retinal pigment epithelium (CHRPE) generally occurs in FAP patients with mutations between codons 311 and 1465. Thus, fully understanding the relationship between phenotype and genotype of FAP could help reveal the nature of disease, predict complications, and further guide clinical treatment.

Here, we report a case of FAP with thyroid cancer as the first presentation and with a negative family history. The mutation of APC is p.Gly977Valfs*3, a genotype that is reported for the first time.

## Case presentation

**Fig. 1:**
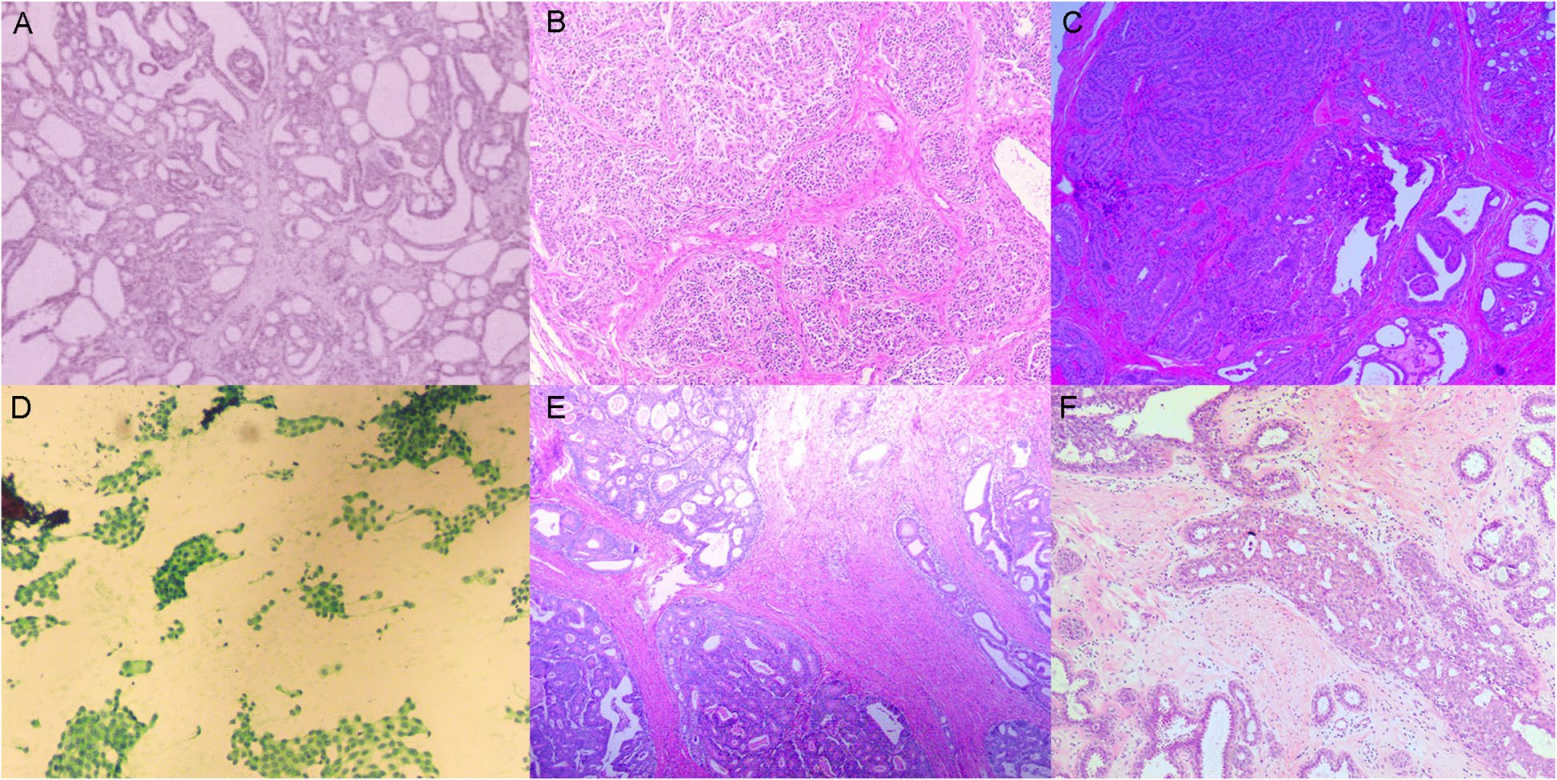
Pathology section images of the patient’s tumor. **A** Primary, **B**, **C** recurrent thyroid cancer, **D** cytological examination of the thyroid, **E** colon cancer, and **F** breast cancer

In 2017, a 20-year-old woman presented to the Department of Thyroid Surgery, The Second Affiliated Hospital of Zhejiang University College of Medicine, Hangzhou, Zhejiang, China, due to an anterior cervical mass. The thyroid ultrasound revealed bilateral nodular hyperplasia of thyroid, and the patient was recommended regular follow-up check. One year later, the patient requested surgical resection for cosmetic purpose. The surgical procedure was changed to total thyroidectomy with prophylactic central node dissection as a papillary thyroid carcinoma was suggested by intraoperative frozen section analysis. The final histopathology report showed bilateral papillary thyroid carcinoma with cystic change (Fig. 1A). Two recurrences presented at 19.0 months and 39.0 months after the initial operation. At the first recurrence, ultrasound suggested multiple lymph node metastases in the neck, and further examination revealed that the patient had liver metastases, with the primary focus in the right colon. Liver magnetic resonance imaging (MRI) scan and diffusion-weighted imaging showed multiple abnormal signal foci in the liver, considered metastases. A suspected ascending colon cancer was detected by MRI. Serum carcinoembryonic antigen (CEA) and alpha-fetoprotein (AFP) levels were within the normal range. Colonoscopy showed multiple polyps of various sizes, with the largest being a cauliflower-like uplift of 3.0-cm diameter in the ascending colon, and pathologically suggested adenocarcinoma (Fig. 1E). The patient underwent laparoscopic right hemicolectomy, laparoscopic lobectomy of the liver, and radiofrequency ablation for two other smaller liver metastases. The pathological diagnosis of the liver neoplasm was adenocarcinoma and immunohistochemistry showed positive CK20 and CDX2 staining, confirming the mass deriving from the large bowel. One regional lymph node was found positive, so the TNM stage (AJCC eighth Edition Staging Manual) was considered pT3N1aM1a. In addition, the hemicolectomy specimen yielded several polyps, all diagnosed as tubular adenomas with low-grade intraepithelial neoplasia. Molecular analysis identified KRAS mutation p.G12V in the carcinoma. The patient was then treated with adjuvant chemotherapy. A XELOX regimen with 200 mg oxaliplatin on day one and 1500 mg capecitabine bid D1–D14 was instituted. After eight cycles of chemotherapy, a colonoscopy 1 year after the resection of the ascending colon adenocarcinoma revealed nearly 100 diffuse polypoid bumps scattered throughout the colon. The three largest polyps were removed and diagnosed as tubular adenomas accompanied by low-grade intraepithelial neoplasia. No obvious abnormalities were observed in the small intestine. About 10 fundic gland polyps were found by gastroscopy in the stomach. The patient suffered a recurrence in hepatic segment VII, and radiofrequency ablation was performed 17 months after the liver operation. Thyroid tumor resection surgery was performed, and papillary thyroid carcinoma was found based on the postoperative pathology report for the first recurrence (Fig. 1B). The second recurrence of thyroid tumor was detected by ultrasound, which found a hypoechoic nodule with a size of 0.88 × 0.61 cm in the left cervical VI region. Fine-needle aspiration (FNA) biopsy was then conducted and cells exhibited nest-like distribution with heteromorphism (Fig. 1D). The operation was then carried out and the pathology revealed papillary thyroid carcinoma (Fig. 1C). In addition, the patient herself found a right breast mass, and pathologically diagnosed as adenosis with intraductal papilloma (Fig. 1F).

Although the patient denied positive family history, she was tested for FAP predisposition with mutational analysis. The result revealed the presence of a frameshift mutation, c.2929delG (p.Gly977Valfs*3) in the APC gene. This mutation has not been recorded in the ClinVar database so far (Table 1), while a deletion site NM_000038.6(APC):c.2928_2929del (p.Gly977fs) (variant ID 1,050,316) close to the detected region was listed in the ClinVar database. Therefore, the mutation detected in this analysis was likely a pathogenic mutation as well.

**Table 1:** The frameshift mutation in c.2929 is related to the deletion of glycine

Gene	Exon	Nucleotide change	Amino acid change	Mutation type	Zygosity
APC	15	c.2929delG	p.G977fs	Frameshift deletion	Heterozygous mutation

## Discussion

FAP syndrome is inherited in an autosomal-dominant manner. However, up to one-third of newly diagnosed cases do not belong to previously identified FAP families. In our case, the patient had a negative family history of FAP, which made diagnosis difficult. Nonetheless, there were still some clues pointing to the development of FAP that will be discussed below.

The clinical characteristics of TC in FAP are different from those in the general population. Studies have revealed that the thyroid tumors in FAP most often are multicentric, bilateral, and tend to occur predominantly in young females [12]. The cribriform-morular variant of papillary thyroid carcinoma (CMV-PTC) is a very rare histological subtype of thyroid carcinoma, accounts for only 0.1–0.2% of all papillary thyroid cancers, and was described for the first time in 1994 as a particular thyroid carcinoma observed especially in patients with FAP [13]. For patients with both CMV-PTC and FAP, approximately half of the patients had thyroid cancer as the first presentation, and more than half of the patients had bilateral cancers with multinodular appearance [14]. In addition, the characteristics of CMV-PTC on ultrasound examination tend to be benign instead of malignant [14]. Although the pathology report of this patient was papillary thyroid carcinoma with cystic changes, the clinical characteristics were similar to CMV-PTC. Therefore, early FNA and colonoscopy for this patient were sensible.

The phenotype of the disease seems to be correlated with the location of the mutation on the APC gene. Most FAP patients with thyroid cancers have germline mutations of the APC gene in exon 15 before codon 1220 [15]. Notably, there is a striking female-to-male ratio of 80:1 in FAP-associated PTC, suggesting that epigenetic factors also play an indispensable role in the development of thyroid cancer in the background of FAP. Cetta et al. [15] reported a woman who had a frame shift mutation in codon 976 of the APC gene, which is close to the mutation site in our case, who also suffered from thyroid cancer at a very young age (18 years old). To the best of our knowledge, the APC locus c.2929 guanine deletion in our study has not been described previously. As a result of this deletion mutation, the DNA coding sequence shifted from “GGT CAA ATG…” to “GTC AAA TGA….” This frameshift results in a mutation of amino acid 977 in the resultant protein product from glycine to valine and further leads to premature translation termination at amino acid 979. The truncated form of APC loses two potential nuclear localization signals (NLS) identified in the C-terminal half of APC, making APC relocate from the nuclei to cytoplasma, which accords with the observation that increases in the cytoplasmic distribution of APC correlate with colon cancer progression [16]. Additionally, the truncated APC protein lacks the 20-amino acid repeats, EB1 binding site, and human disc large (HDLG) binding site. β-catenin and axin bind to APC at the 20-amino acid repeat motifs in a complex that promotes GSK3β-mediated phosphorylation of serine and threonine residues of β-catenin [17]. β-catenin is therefore marked for subsequent degradation by ubiquitin-mediated proteolysis and thus suppresses tumorigenesis [18, 19]. EB1 is involved in the regulation of microtubule function during the cell cycle; The absence of its binding domain on APC may be associated with indiscriminate microtubule binding [17, 20]. The HDLG is a tumor suppressor protein. The APC–HDLG complex seems to take the responsibility for disturbing progression of the cell cycle from G0/G1 to S phase. Thus, loss of the HDLG binding site on APC may result in decreased regulation of cell cycle [17].

### Clinical characteristics

Thyroid cancer in a patient with FAP was first reported by Crail in 1949 [21]. After that, the understanding of thyroid cancer as an extra-colonic manifestation has gradually become more recognized [22]. Plail et al. [23] analyzed 998 FAP patients and reported that young women (less than 35 years of age) with adenomatosis polyposis are approximately 160 times more likely to develop thyroid cancer than the general population. The incidence of thyroid cancer among FAP patients is still disputable. Many studies have indicated that the incidence of thyroid carcinoma in FAP patients is between 1 and 2% [24, 25], but this is considered an underestimation [26]. Surprisingly, Herraiz et al. [27] reported a significantly higher prevalence (12%) of TC in 51 FAP patients. It is inappropriate to simply consider the study too small to draw a conclusion as smaller sample size often means a more thorough examination. A meta-analysis in 2019 enrolling 9821 patients suggested that the prevalence of TC in FAP patients was about 2.6%, whereas the prevalence of TC in the general population was reportedly 0.14% [4]. In the meantime, studies that used screening ultrasound, with study populations < 200, and published after 2002, had a significantly higher prevalence of TC [4]. This may be attributed to (a) a considerable part of detected TC depended on the presence of clinical symptoms for diagnosis and old studies may not perform systematic examinations for every patient; (b) the ultrasound manifestations of CMV-PTC tend to be benign, leading to easy missed diagnosis; (c) the survival benefit from prophylactic colectomy makes the extracolonic manifestations more frequent. Therefore, the incidence of FAP-associated thyroid cancer is probably larger than previously thought. The case in our study is typically a young female, as there was a striking female to male ratio of 19:1, suggesting that epigenetic or environmental factors (such as hormonal factors) are involved in TC occurrence in the context of FAP [4]. In addition, whereas the prevalence of sporadic thyroid cancer peaks when people are in their 60 s, it develops in patients with FAP at a much younger age (about 30 years old). For patients with both CMV-PTC and FAP, approximately half of the patients had thyroid cancer as the first presentation, and more than half of the patients had bilateral cancers with multinodular appearance [14].

### Genotype–phenotype correlation

Concerning genotype–phenotype correlations, we reviewed articles focused on FAP-associated TC and published in the past 10 years. As summarized in Table 2, in accordance with the past recognition, most mutations are found before codon 1220 [15], but there are also some exceptions. For example, in FAP-TC patients, mutations in codon 1309 were reported by many researchers [15, 28–30]. However, this mutation is the most common APC mutation in FAP patients and when adjusting for the reference population prevalence, mutations in codon 1309 were actually less common as a percentage of the total in the FAP-TC group than in the FAP reference group [31]. In addition, mutations in codons 1275, 1307, 1394, 1465, and 2092 were also reported [28, 30, 32]. Septer et al. [31] compared the prevalence of APC mutations in the population of FAP patients with thyroid cancer and the prevalence of the same mutation in the unselected FAP population and concluded that there was an increased risk of thyroid cancer in individuals with APC mutations at the 5′ end (proximal to codon 528) along with the established high-risk group harboring mutation at codon 1061. Although progresses have been made in identifying the relationship between the development of thyroid cancer and specific mutant regions, TC may occur in patients with germline mutations throughout the APC gene. Additionally, Cetta et al. [33] reported 3 siblings with FAP-associated PTC, one patient showed the typical CMV-PTC variant, whereas the other 2 sisters showed the “usual” papillary variant despite having the same germline APC mutation. It was previously considered that TC was closely associated with CHRPE since most of the mutations clustered in the same genomic area, whereas desmoid tumors were much less associated because the common mutation site was after codon 1444. Nevertheless, Truta et al. [28] reported that 6 of 16 FAP patients with thyroid cancer also developed desmoid tumors; in 5 of these cases, the genetic APC mutations were identified before codon 1444. In summary, germline mutations in specific regions of APC gene may confer only a genetic susceptibility but not an absolute destiny for the occurrence of TC.

**Table 2:** Summary of APC germline mutations in FAP-TC patient cases reported in the literature

**Codon no./mutation (# of patients)**	**Number of patients**	**Recurrence (# of patients)**	**CHRPE (# of patients)**	**Other ECM**	**Authors, year/references**
Codon 848 (2)	2	NR	Yes (2)	Desmoids; duodenal polyposis	H Kashiwagi, 1996 [39]
Codons 313 (2), 698 (3)	5	Yes (2)	NR	endometrial cancer; R maxilla osteoma; UGI polyposis	Soravia C, 1999 [37]
Codon 1110 (TCA to TGA), 175 (C deletion)	2	NR	Yes (1)	NR	Iwama T, 1999 [40] Miyaki M, 2000 [34]
Codons 140, 593, 778, 976, 993, 1061 (*n* = 5), 1105, and 1309 (*n* = 2)	13	NR	Yes (11)	Hepatoblastoma	F. Cetta, 2000 [15]
Codon 1061, 5-bp deletion at position 3183	1	Yes	Yes	Medulloblastomas; duodenal polyps	Fenton PA, 2001 [41]
Codons 159, 499, 1–804, 937–938, 686–1217, 1061 (2), 1068, 1275, 1309, 2092 (2)	12	NR	Yes (2)	UGI polyps; desmoid; sebaceous cyst; epidermoid cyst; lipoma; osteoma	Truta B, 2003 [28]
Codon 512	1	NR	NR	NR	Xu B, 2003 [35]
Codon 278, (CAG to TAG, stop)	2	NR	NR	NR	Kameyama K, 2004 [42]
Codon 1309: five nucleotides (AAAGA) were deleted	2	NR	Yes (1)	Stomach polyps; ampullar ulcer	Lee S, 2004 [29]
Codon 554, CGA[Arg] to TGA[Stop]	1	NR	NR	NR	Uchino S, 2006 [43]
heterozygous APC Ex 2–3 duplication mutation	1	Yes	No	Lung metastases; brain metastases	Cameselle-Teijeiro J, 2009 [38]
c.3183_87delACAAA; del9-10 (del9080dup11) (3)	4	NR	NR	NR	A Martayan, 2010 [44]
Codon 332 (R332X)	1	No	None	None	Steinhagen E, 2012 [45]
Codons 917 T deletion; 1179–11,817-bp deletion; 1483 C deletion	3	NR	NR	NR	Kumamoto K, 2015 [46]
Codons 159 (*n* = 2), 161, 302, 689, 964, 1068, and 1157, 1 deletion at APC locus	9	NR	NR	NR	Uchino S, 2016 [47]
Codon737 p.(Tyr737*)	1	NR	NR	NR	Akaishi J, 2018 [48]
Codon 935	1	No	No	Bilateral breast fibromatosis in the context of silicone prosthetics	Silva S, 2018 [49]
Codons 654, 769, 834, 1062, 1073, 1394	6	NR	NR	NR	Park J, 2019 [32]
Stop codon at codon 325	1	Yes	NR	NR	Ito Y, 2019 [50]
Codons 213 (2 from the same family (c.637C > T)), 1062 (2 from the unrelated family (c.3183_3187delACAAA))	4	NR	NR	NR	de Oliveira JC, 2019 [51]
Codons 471, 578, 582, 625, 704, 1062, 1068 (2), 1307, 1309, 1465, full gene deletion	12	NR	NR	Desmoid tumors; adrenal adenoma Ampullary cancer	Nieminen TT,2020 [30]

*CHRPE* Congenital hypertrophy of the retinal pigment epithelium, *ECM* Extracolonic manifestations, *NR* Not reported, *UGI* Upper gastrointestinal

An APC germline mutation results in the inactivation of only one allele, and there is still a residual APC-gene that can compensate, at least partly, for the loss of function. Therefore, in the context of pathogenic germline mutations of APC, the remaining somatic APC mutation or other phenotypic-equivalent mutations can lead to tumorigenesis. Interestingly, two different somatic inactivating APC variants have been detected in a sporadic case of CMV-PTC without APC germline mutation, confirming the above speculation [8]. The occurrence of multiple carcinomas in one thyroid gland is frequently observed in FAP patients. Miyaki and coworkers noted that each carcinoma had a different somatic mutation of the APC gene, suggesting independent development of multicentric thyroid carcinomas in FAP patients [34]. Moreover, Nieminen et al. [30] reported that six of seven patients harboring both germline and somatic APC mutations exhibited PTC-CMV histology, and the only one who had the conventional papillary thyroid cancer harbored the mutation at codon 1726. The explanation may be that the mutated APC protein in this patient retained some beta-catenin binding ability. Thus, the somatic mutations may also have an association with the histology of the tumor. In addition to APC, mutations in CTNNB1 (β-catenin) and AXIN1 could lead to the same outcome through a similar mechanism. β-catenin binds to APC and axin in a complex that promotes GSK3β-mediated phosphorylation of its own serine and threonine residues. In this way, β-catenin is marked for subsequent degradation by ubiquitin-mediated proteolysis [17]. Mutations in CTNNB1 result in attenuated β-catenin phosphorylation with the ultimate consequence of reduced β-catenin degradation and the subsequent unregulated activation of the WNT pathway. Axin thus acts as a negative regulator of the wnt-signaling pathway by reducing the amount of β-catenin available for transcriptional activation [17]. Xu et al. reported mutations in exon 3 of the beta-catenin gene (CTNNB1) in seven tumors from both familial and sporadic CMV-PTC patients [35]. AXIN1 somatic mutations (exons 1 and 7) have also been reported in a case of CMV-PTC [36]. Moreover, it is found that somatic mutations in KMT2D and KMT2C are highly frequent in FAP-TC, while mutations in these two genes are rarely detected in sporadic TC [30]. Further investigation showed that KMT2D together with the ALK gene were connected with CTNNB1; thus, the KMT2D might also be involved in WNT signaling and promote tumorigenesis [30].

The RET/PTC rearrangement, a molecular alteration associated with sporadic papillary thyroid cancer, was also observed in some FAP-associated TC cases [37], including one with aggressive behavior [38]. It is considered that the loss of function of the APC gene is associated with gain of function of RET/PTC in FAP-associated thyroid cancer [37].

### Prognosis

CMV-PTC generally has an indolent character and distant metastases are extremely rare in patients with CMV-PTC. To the best of our knowledge, only 5 patients with CMV-PTC had distant metastases (Table 3), three were FAP-associated and two were sporadic. In three FAP-associated CMV-PTC cases, APC germline mutations are a 5-bp deletion at position 3183 (codon 1061), a heterozygous APC Ex 2–3 duplication, and a premature stop codon at codon 325, respectively [38, 41, 50]. In two sporadic cases, somatic mutations are APC p.Cys520Tyr_fsX534 and a TERT promoter mutation, respectively [52, 53]. Cameselle-Teijeiro et al. [38] reported a case of aggressive CMV-PTC with neuroendocrine differentiation harboring both an APC germline mutation (heterozygous APC Ex 2–3 duplication) and somatic mutations (APC homozygous silent p.Thr1493Thr gene variant; RET/PTC rearrangement). The patient died of lung and brain metastases only 17 months after thyroidectomy. The tumor was positive for chromogranin and synaptophysin while these markers were negative in another case of FAP-associated aggressive CMV-PTC [50]. Although it is hard to draw a conclusion due to the small number of reported cases, some characteristics could be found: (a) Patients with metastatic CMV-PTC tend to have a later age of onset. (b) Either unusual histological or molecular features were seen in three of the five patients. (c) In FAP-associated patients, the germline mutation sites of the APC gene are consistent with the region associated with thyroid cancer.

**Table 3:** Summary of cases with metastatic CMV-PTC

APC germline mutation	Somatic mutation	Gender, age at diagnosis	FAP association	Lymph node metastasis	Distant metastasis (age)	Other tumors	Histological type	Reference
A 5-bp deletion at position 3183 (codon 1061)	None	F, 20	FAP-associated	None	Bone (50)	Medulloblastoma (age 6)	Mixed follicular carcinoma and medullary thyroid carcinoma, positive staining for calcitonin	[41]
Heterozygous *APC* Ex 2–3 duplication	APC homozygous silent p.Thr1493Thr gene variant; *RET/PTC* rearrangement	M, 42	FAP-associated	None	Lung; brain (43)	Osteomas	Cribriform-morular variant of papillary thyroid carcinoma, showing neuroendocrine differentiation	[38]
None	APC p.Cys520Tyr_fsX534	F, 35	sporadic	None	Lung; brain (35)	None	CMV-PTC and Poorly differentiated thyroid carcinoma (PDTC)	[52]
None	*TERT* promoter mutation	F, 45	sporadic	5/23	Bone	None	CMV-PTC	[53]
APC gene mutation (stop codon at codon 325)	Not reported	F, 24	FAP-associated	None	Lung (27)	None	CMV-PTC	[50]

### Surveillance

Ultrasound screening for TC in FAP patients remains controversial and a guideline for FAP-associated TC screening has not been established yet. Feng et al. [54] showed that patients with TC who had thyroid ultrasound screening (*n* = 15) may possess smaller tumor size and consequently require less radical therapy. However, neither the rate of complications nor the recurrence/metastases showed a significant difference. The American College of Gastroenterology recommended patients with FAP annual thyroid ultrasound screening. However, the evidence that supports this recommendation is insufficient [55]. According to the American Thyroid Association guidelines for early diagnosis of TC in FAP, the use of ultrasound would lead to a reduction of morbidity and mortality, but this is based on insufficient evidence as well [56]. Considering the low clinical benefit, a review focused on thyroid cancer screening supported systematic neck palpation at the outpatient clinic and that ultrasound screening could be considered in a clinical research setting for patients with hereditary syndromes including FAP [57]. Given the potential harms for ultrasound screening such as psychological issues, decreased quality of life, financial issues, and high false-positive rates due to its low specificity (55% [58]), we agree with the opinion of Chenbhanich and co-workers that FAP patients with high-risk factors for TC (such as a young female with an APC mutation proximal to the 5′ end) rather than every FAP patient should receive thyroid ultrasound screening [4]. However, larger scales of research and longer time of follow-up are required to determine whether such screening has an impact on TC survival in FAP patients.

## Conclusion

According to the abovementioned mechanisms and the clinical manifestations of the patient, we can conclude that mutation c.2929delG(p.Gly977Valfs*3) in exon 15 of the APC gene is a newly identified pathogenic mutation. Our study enriched the APC mutation spectrum and deepened the insights on FAP, which may help to facilitate early identification and treatment of FAP patients.

## Data Availability

All data generated during this study are included in this published article.

## Abbreviations

FAP: Familial adenomatous polyposis
CRC: Colorectal cancer
APC: Adenomatous polyposis coli
TC: Thyroid cancer
CMV-PTC: Cribriform-morular variant of papillary thyroid carcinoma
CHRPE: Congenital hypertrophy of the retinal pigment epithelium
